# Two cases of parathyroid carcinoma associated with multiple brown tumours

**DOI:** 10.1093/bjrcr/uaad003

**Published:** 2023-12-13

**Authors:** Akihiro Sakai, Toshihide Inagi, Hiroaki Iijima, Koji Ebisumoto, Kenji Okami

**Affiliations:** Department of Otolaryngology, Head and Neck Surgery, Tokai University, School of Medicine, Isehara, Kanagawa 259-1193, Japan; Department of Otolaryngology, Head and Neck Surgery, Tokai University, School of Medicine, Isehara, Kanagawa 259-1193, Japan; Department of Otolaryngology, Head and Neck Surgery, Tokai University, School of Medicine, Isehara, Kanagawa 259-1193, Japan; Department of Otolaryngology, Head and Neck Surgery, Tokai University, School of Medicine, Isehara, Kanagawa 259-1193, Japan; Department of Otolaryngology, Head and Neck Surgery, Tokai University, School of Medicine, Isehara, Kanagawa 259-1193, Japan

**Keywords:** parathyroid carcinoma, multiple brown tumours, bone metastasis

## Abstract

We present two rare cases of parathyroid carcinomas associated with multiple brown tumours. Plain radiographs, computed tomography, and neck ultrasonography revealed the presence of bone and parathyroid tumours. Despite the use of 99m Tc-methoxy isobutyl isonitrile (99mTc-MIBI) or ^18^F-fluorodeoxyglucose-positron emission tomography (18F-FDG PET)/CT, it was difficult to differentiate bone metastases from brown tumours. Parathyroid carcinoma was confirmed by histopathological examination following parathyroidectomy, resulting in spontaneous bone lesion improvement. In patients with parathyroid carcinoma presenting with bone lesions suggestive of metastasis, understanding the potential for brown tumour accumulation through 99mTc-MIBI or 18F-FDG PET/CT is pivotal. With this understanding, it is possible to diagnose brown tumours with parathyroidectomy and follow up for improvement of bone lesion and avoid invasive biopsy or surgery.

## Introduction

Parathyroid carcinoma is a rare endocrine malignancy that arises from the parathyroid glands and is a rare cause of hyperparathyroidism, accounting for less than 1% of cases.^1^^,^^2^ Owing to its rarity, the diagnosis of parathyroid carcinoma is often delayed, and its presentation can vary. Parathyroid carcinomas can present with primary hyperparathyroidism^3^ or symptoms related to local invasion, or distant metastases.^4^ The diagnosis of parathyroid carcinoma is based on histopathological examination, and the treatment of choice is surgical resection.^1^^,^^4^^,^^5^

Brown tumours, also known as osteitis fibrosa cystica, are focal bone lesions that develop in patients with longstanding hyperparathyroidism.^6^ These tumours occur due to increased osteoclastic activity resulting from elevated levels of parathyroid hormone (PTH), leading to bone resorption and subsequent fibrous tissue replacement. Although brown tumours are typically benign, they can mimic malignant bone tumours.^6^ In other words, when multiple brown tumours are found, parathyroid carcinoma metastasis may not be ruled out.^3^^,^^7^ Although the association between brown tumours and hyperparathyroidism is well known, reports of brown tumours associated with parathyroid carcinoma are rare.^7^^,^^8^ Herein, we present two cases of parathyroid carcinoma with brown tumours and emphasize the need to consider parathyroid carcinoma in the differential diagnosis of patients with brown tumours.

## Case presentation

### Case 1

A 65-year-old man presented to our hospital with a chief complaint of pain in the right elbow. He was previously diagnosed with a right olecranon tumour and a pathological fracture of the olecranon of the right ulna by plain radiographs. However, bone scintigraphy revealed multiple bone accumulations throughout the body, and the patient was referred to our department because of a suspicion of bone metastasis of the cancer.

**Figure 1.**
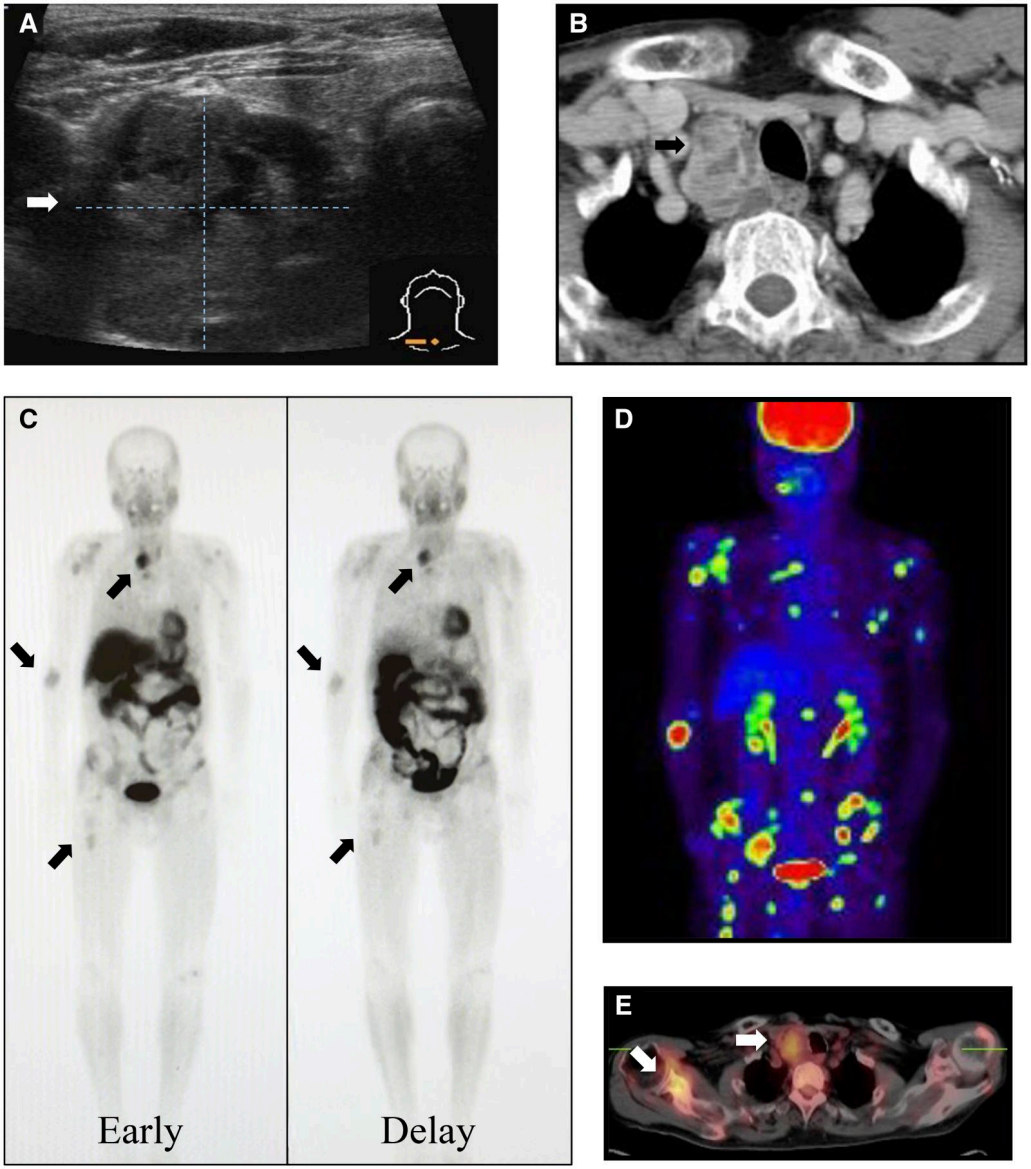
Neck ultrasonography (A) and contrast-enhanced computed tomography (B) show an internally heterogeneous mass at the lower pole of the right lobe of the thyroid gland with an indistinct boundary with the surrounding tissue. 99mTc-MIBI scintigraphy (C) shows abnormal accumulation in the superior mediastinum and multiple bone. 18F-FDG PET/CT also shows a weak accumulation in the superior mediastinum (D) and a strong accumulation of multiple bone (E). 18F-FDG PET/CT = ^18^F-fluorodeoxyglucose-positron emission tomography/computed tomography; 99mTc-MIBI = 99m Tc-methoxy isobutyl isonitrile.

In the biochemical findings, calcium was elevated at 14.1 mg/dL (normal range: 8.8-10.1 mg/dL), and intact PTH (iPTH) was elevated at 1940 pg/mL (normal range: 10-65 pg/mL). Neck ultrasonography and CT revealed a heterogeneous internal mass extending from the inferior pole of the right lobe of the thyroid gland to the upper mediastinum (Figure 1A and B). The boundary between the thyroid gland, oesophagus, and trachea remains unclear. 99m Tc-methoxy isobutyl isonitrile (99mTc-MIBI) scintigraphy and ^18^F-fluorodeoxyglucose positron emission tomography (18F-FDG PET)/CT revealed multiple bone accumulations (Figure 1C and D) and a weak accumulation in the superior mediastinum (Figure 1E).

The patient was diagnosed with suspected parathyroid carcinoma with brown tumours or metastasis of the parathyroid carcinoma. The treatment plan included the removal of the parathyroid tumour, follow-up observation of bone lesions, and bone biopsy, if necessary.

**Figure 2.**
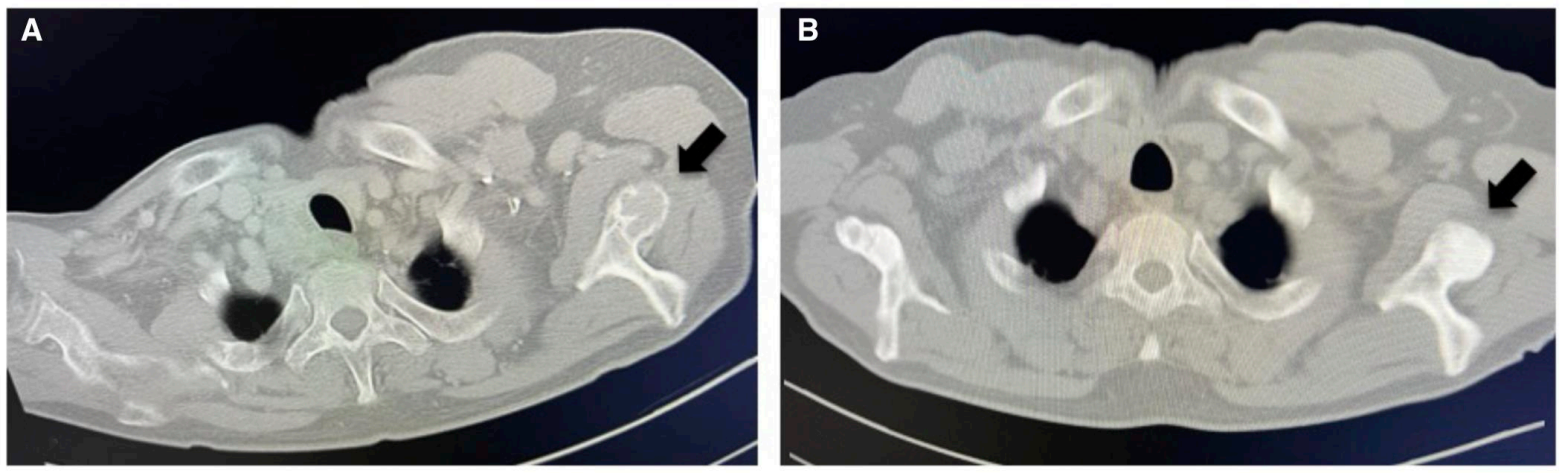
Osteolytic lesions of the scapula preoperatively (A) show sclerotic changes at 6 months postoperatively (B).

The patient’s iPTH levels normalized to 15 pg/mL the day after surgery and maintained in the normal range thereafter. Bone density normalized from 58% to 96% at 713 days postoperatively. The final pathological diagnosis was a parathyroid carcinoma. CT showed sclerotic changes in the bilateral rib and scapular osteolytic lesions at 6 months postoperatively (Figure 2), indicating a high possibility of a brown tumour. Calcium gluconate (435 mg/A) was administered intravenously at a maximum daily dose of 30 A and tapered off over approximately 1 month. Simultaneously, oral calcium gluconate at a maximum daily dose of 21.6 g was also started and gradually reduced over approximately 700 days. Currently, more than 10 years after surgery, there has been no evidence of recurrence or metastasis.

### Case 2

**Figure 3.**
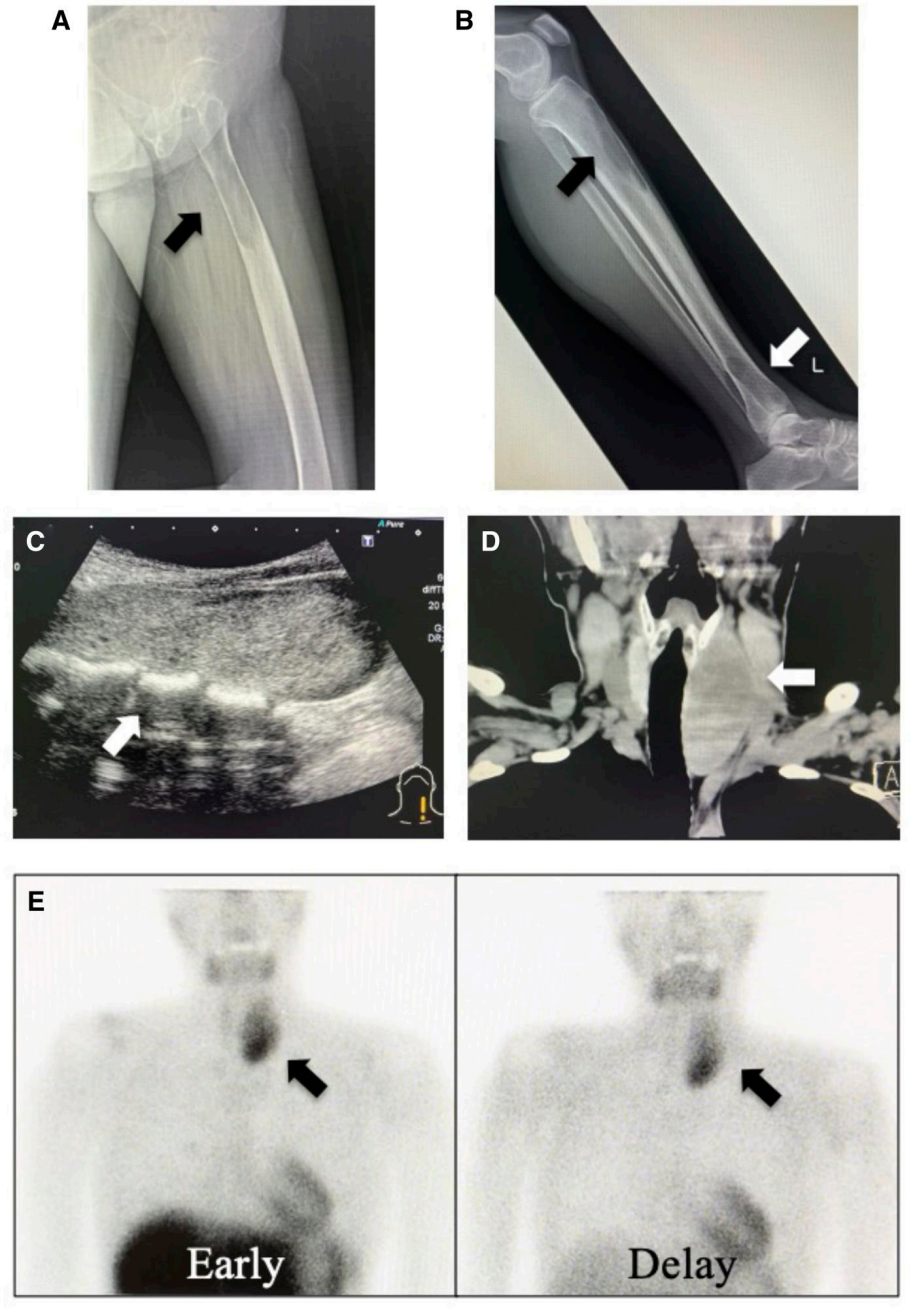
Plain radiographs of the femur (A) and tibia (B) show multiple osteolytic lesions. Neck ultrasonography (C) and computed tomography (D) show an 80 mm tumour behind the left lobe of the thyroid gland. 99mTc-MIBI scintigraphy (E) shows abnormal accumulation in the superior mediastinum. There is no accumulation in the bone lesions. 99mTc-MIBI = 99m Tc-methoxy isobutyl isonitrile.

A 46-year-old woman presented to our hospital with hip pain. She was diagnosed with a left femoral facet fracture secondary to a bone tumour. Gamma nail insertion for fracture prevention and bone biopsy were performed. Plain radiographs revealed multiple osteolytic changes in the clavicle, scapula, ribs, spine, pelvis, and femur (Figure 3A and B). Neck ultrasonography and CT revealed an 80 mm low-absorption mass in the dorsal left lobe of the thyroid gland (Figure 3C and D). 99mTc-MIBI showed abnormal accumulation in the dorsal left lobe of the thyroid gland but no accumulation in the bone lesions. The patient’s blood test showed abnormal Ca levels (14.4 mg/dL) and iPTH (931 pg/mL). The pathological findings of the bone biopsy showed osteoclast proliferation, bone resorption, and hemosiderin deposition. Based on these results, the primary tumour was diagnosed as a parathyroid tumour, and the bone lesions were diagnosed as brown tumours.

**Figure 4.**
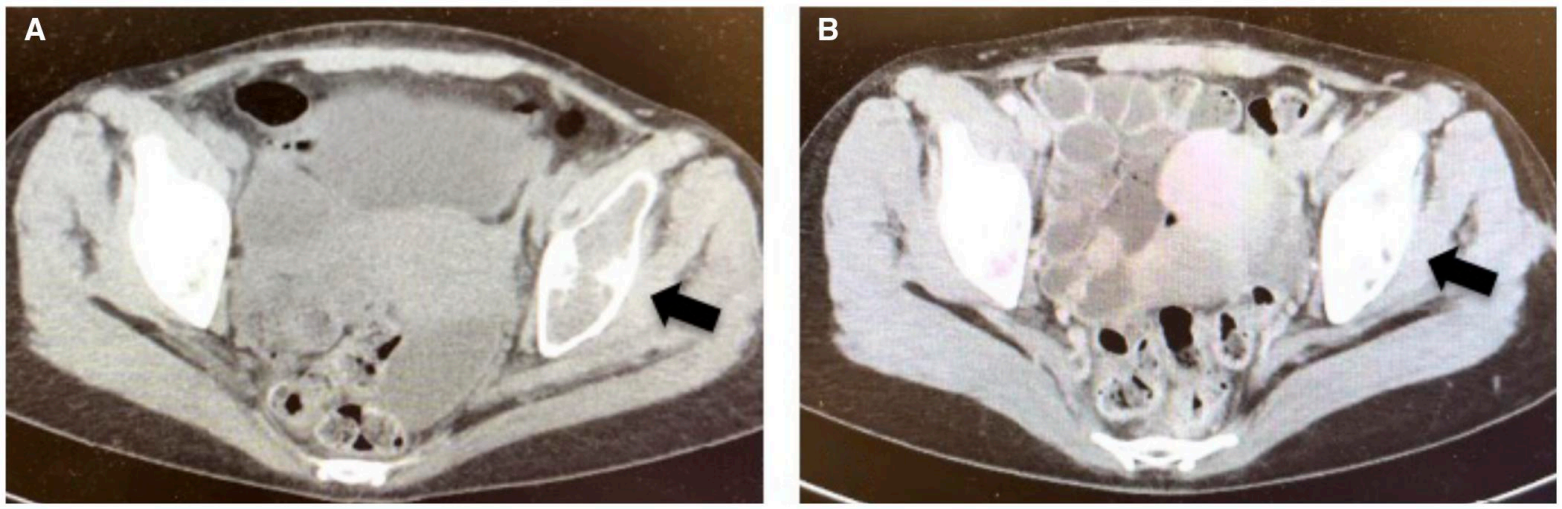
Osteolytic lesions of the pelvis preoperatively (A) show sclerotic changes at 6 months postoperatively (B).

Parathyroidectomy was performed. Pathological findings revealed parathyroid carcinoma. The iPTH level decreased to 64 pg/dL the day after surgery and normalized to 25 pg/dL 1 year later. CT showed sclerotic changes in the pelvic osteolytic lesion 6 months postoperatively (Figure 4A and B). The patient's bone density in the left femur increased from 59% to 70% at 383 days postoperatively. Calcium gluconate was administered intravenously at a maximum daily dose of 10 A, and oral calcium gluconate at a maximum daily dose of 25.2 g was also administered for approximately 200 days. Currently, 2 years after surgery, there has been no evidence of recurrence or metastasis.

The summary of cases 1 and 2 is shown in Table 1.

**Table 1.** Summary of cases 1 and 2.

Variables	Case 1	Case 2
Age	65	69
Sex	Male	Female
Chief complaint	Right elbow pain	Hip pain
Initial diagnosis	Right elbow tumour	Left femoral fracture
Biopsy of bone	–	Brown tumour
Tumour size	50 mm	82 mm
99mTc-MIBI		
Parathyroid	Positive	Positive
Bone	Positive	Negative
18F-FDG PET/CT		
Parathyroid	Positive	–
Bone	Positive	–
Pathological diagnosis	Parathyroid carcinoma	Parathyroid carcinoma
Calcium (normal range: 8.8-10.1 mg/dL)	14.1	14.4
iPTH (normal range: 10-65 pg/mL)	1940	931
Postoperative iPTH	15	64
Bone densitometry	Lumbar: 58 → 96%（POD713）	Femoral: 59 → 92%（POD383）

Abbreviations: 18F-FDG PET/CT = ^18^F-fluorodeoxyglucose positron emission tomography/computed tomography; iPTH = intact parathyroid hormone; 99mTc-MIBI = 99m Tc-methoxy isobutyl isonitrile; POD = postoperative day.

## Discussion

In this study, two patients with parathyroid carcinoma were identified following the detection of brown tumours. These tumours can be difficult to differentiate from primary bone tumours or metastatic bone lesions because they can be depicted as osteolytic or neoplastic lesions on plain radiographs or CT. Case 1 was initially suspected of having bone metastasis of parathyroid carcinoma. This was because 99mTc-MIBI and 18F-FDG PET/CT showed multiple bone accumulations, and a clinical suspicion of parathyroid carcinoma was made.

99mTc-MIBI is a specific imaging modality used to evaluate the parathyroid glands. It accumulates in the thyroid gland and enlarges the parathyroid gland during early phases. In the delayed phase, accumulation in the normal thyroid gland disappears, which is helpful for the localization diagnosis of parathyroid carcinoma. Whilst parathyroid carcinomas and bone metastasis often show accumulation,^5^^,^^9^ brown tumours are considered less likely to accumulate.^10^ However, there have been some reports of its accumulation in tumours,^3^ and in such cases, differentiation from metastasis becomes difficult. 18F-FDG PET/CT is considered highly effective for the diagnosis of parathyroid carcinoma.^11^ In contrast, brown tumours, despite being benign, often accumulate and should be treated with caution.^2^^,^^6^^,^^7^ In other words, the typical finding is that bone metastases show accumulation in both 99mTc-MIBI and 18F-FDG PET/CT, whilst brown tumours show accumulation in 18F-FDG PET/CT and are negative in 99mTc-MIBI.

In case 2, 99mTc-MIBI accumulated only in the parathyroid gland and not in the bone lesions. Unfortunately, a preoperative 18F-FDG PET/CT scan could not be performed because it was not covered by insurance. However, based on the finding that the multiple bone lesions seen on X-ray and CT were not accumulated on 99mTc-MIBI, it was possible to diagnose that the bone lesions were brown tumours.

In contrast, in case 1, 99mTc-MIBI accumulated in both parathyroid and bone lesions. In addition, 18F-FDG PET/CT showed accumulation in both parathyroid and bone lesions. Thus, when a bone lesion accumulates on 99mTc-MIBI and 18F-FDG PET/CT, differentiating between brown tumours and bone metastases becomes challenging. The mechanism of increased 99mTc-MIBI uptake in brown tumours has not yet been elucidated, but is considered to result from increased perfusion, metabolism, and osteoclast activity.^12^ Meng et al. reported that a high level of PTH was associated with a higher rate of positivity in 99mTc-MIBI for brown tumours.^10^ In case 1, the level of intact PTH was extremely high at 1940 pg/mL. Therefore, the MIBI uptake could have been observed.

Clinical findings suggestive of parathyroid carcinoma have been reported as follows: palpable cervical mass, serum Ca level ≥12 mg/dL, iPTH level ≥300 pg/mL, and bone lesions other than osteoporosis.^4^ In both patients, the clinical findings were suggestive of parathyroid carcinoma, which may have made the diagnosis of bone lesions even more difficult owing to the suspicion of carcinoma.

Brown tumours are often diagnosed by biopsy of bone lesions to exclude malignancy.^6^ In this study, case 1 was diagnosed without biopsy, whereas case 2 was diagnosed by biopsy. Once brown tumours are diagnosed, no specific treatment is necessary. Resection of the parathyroid tumour promotes gradual sclerotic changes in the bone lesion and 18F-FDG PET/CT accumulation disappears.^7^ In addition, rapid normalization of iPTH levels after parathyroidectomy and the presence of postoperative hungry bone syndrome are also important findings suggestive of brown tumours.^7^^,^^8^ In these two cases, the iPTH level normalized immediately and the bone lesions improved spontaneously after the resection of the parathyroid tumours. Furthermore, the metastatic sites of parathyroid carcinoma are reported to be the cervical lymph nodes (30%), lungs (40%), and liver (10%), with bone metastasis being less frequent. In a review by Alberti et al.,^5^ there were 3 out of 20 cases with bone metastasis only. In other words, cases of bone metastasis alone without other sites of metastasis may be extremely rare.

In patients with parathyroid carcinoma and bone lesions suspected of metastasis, adequate knowledge of hyperparathyroidism, parathyroid carcinoma, and brown tumours may allow the patient to avoid invasive biopsy or surgery by performing parathyroid surgery and follow-up of bone lesions.

In conclusion, when diagnosing a patient with a parathyroid tumour and a bone lesion that accumulates on 18F-FDG PET/CT or 99mTc-MIBI, brown tumour should be mentioned as a differential diagnosis as well as bone metastasis. The most important point is the knowledge that brown tumours can accumulate on 18F-FDG PET/CT or 99mTc-MIBI despite their benign nature. With this knowledge, it would be possible to diagnose the bone lesion as a brown tumour from the course of the disease by first performing parathyroidectomy, confirming an immediate decrease in iPTH, followed by imaging of the bone lesion. In this way, invasive biopsy or surgery could be avoided.

## Learning points

- Brown tumours, associated with hyperparathyroidism, can mimic malignant bone tumours, posing diagnostic challenges.
- Both 99mTc-MIBI and 18F-FDG PET/CT imaging modalities can contribute valuable information in diagnosing parathyroid carcinoma. However, if these imaging scans show multiple accumulations in the bone, the possibility that it is not just a bone metastasis but also a benign brown tumour should be considered.
- Understanding the possibility of brown tumour accumulation on 99mTc-MIBI or 18F-FDG PET/CT, the diagnosis of a brown tumour can be confirmed by first performing parathyroidectomy and then monitoring the clinical course. This avoids unnecessary invasive biopsy or surgery for bone lesions.
